# Engineering Plasmonic Environments for 2D Materials and 2D-Based Photodetectors

**DOI:** 10.3390/molecules27092807

**Published:** 2022-04-28

**Authors:** Jianmei Li, Jingyi Liu, Zirui Guo, Zeyu Chang, Yang Guo

**Affiliations:** 1 State Key Laboratory of Metastable Materials Science and Technology & Key Laboratory for Microstructural Material Physics of Hebei Province, School of Science, Yanshan University, Qinhuangdao 066004, China; jingyiliuysu@yeah.net (J.L.); ziruiguoysu@yeah.net (Z.G.); zeyuchang@yeah.net (Z.C.); 2 Beijing National Laboratory for Condensed Matter Physics, Institute of Physics, Chinese Academy of Sciences, Beijing 100190, China; 3 School of Physical Sciences, CAS Key Laboratory of Vacuum Physics, University of Chinese Academy of Sciences, Beijing 100190, China

**Keywords:** plasmonic nanostructure, metamaterials, Purcell effect, two-dimensional materials, photodetector

## Abstract

Two-dimensional layered materials are considered ideal platforms to study novel small-scale optoelectronic devices due to their unique electronic structures and fantastic physical properties. However, it is urgent to further improve the light–matter interaction in these materials because their light absorption efficiency is limited by the atomically thin thickness. One of the promising approaches is to engineer the plasmonic environment around 2D materials for modulating light–matter interaction in 2D materials. This method greatly benefits from the advances in the development of nanofabrication and out-plane van der Waals interaction of 2D materials. In this paper, we review a series of recent works on 2D materials integrated with plasmonic environments, including the plasmonic-enhanced photoluminescence quantum yield, strong coupling between plasmons and excitons, nonlinear optics in plasmonic nanocavities, manipulation of chiral optical signals in hybrid nanostructures, and the improvement of the performance of optoelectronic devices based on composite systems.

## 1. Introduction

The discovery of atomically thin graphene nearly 20 years ago [1] has led to the rapid development of two-dimensional (2D) layered materials. To date, a series of new 2D materials have emerged and been predicted [2,3]. Thanks to the excellent characteristics of these 2D materials, they have served as a promising and competitive family of materials for photonic and optoelectronics [4,5,6]. First, compared with traditional materials, all of these 2D materials can achieve arbitrary stacking of different materials without considering lattice matching because of interlayer van der Waals (vdW) interactions [7]. This enables the construction of diverse 2D material heterostructures [8] or the integration of photonic environments with 2D materials [9,10,11,12,13]. Second, the family of 2D materials has abundant members and continuously spans from insulators and semiconductors to semimetals [14]. Thus, their photo responses cover a wide range of electromagnetic spectra from ultraviolet to infrared [7,14]. Third, some 2D materials, especially monolayer transition metal dichalcogenides (TMDs), are direct bandgap semiconductors [15,16,17] with extremely large exciton-binding energy at room temperature (RT), exhibiting greatly enhanced photoluminescence (PL) intensity [17] which will be beneficial to the development of high-efficiency light-emitting devices. Fourth, some of the 2D materials exhibit high carrier mobility [18,19] at RT which meet the requirements of fast carrier extraction or photoresponse for photonic and optoelectronic devices. Finally, all of these 2D materials have advantages of transparency, flexibility, and ease of fabrication [20] that provide new opportunities for wearable optoelectronic devices.

However, the absolute light absorption of 2D materials is very low, due to their atomically thin thickness that leads to low photo capture efficiency and a low generation rate of photogenerated carriers. Therefore, efficiently enhancing the light–matter interaction is highly desirable for photonic and optoelectronic devices based on 2D materials. A promising approach is to engineer the plasmonic environment around 2D materials for modulating light–matter interaction in 2D materials [5,6,21,22,23,24,25,26,27,28,29,30,31,32]. This method greatly benefits from the advances in the development of nanofabrication and vdW interaction of 2D materials. A plasmonic environment constructed of metal nanostructures has the characterizations of subwavelength optical localization and enhanced intensity of light [33,34,35,36] due to the excitation of surface plasmons (SP) inside it by lights. Efficiently engineering a plasmonic environment can greatly enhance the light–matter interaction in 2D materials which can modulate photo capture, light emission, photocurrent, and nonlinear optics of TMDs materials. Aiming to efficiently use the excellent characteristics of 2D materials apart from the plasmonic environment, 2D materials also can be combined with other nanostructures such as quantum dots [37], carbon nanotubes, and 2D III-V semiconductors to form hybrid heterojunctions [38] which have the potential to markedly enhance absorption and quantum efficiency properties.

In this article, we summarize the advancement of the frontier in 2D material coupled with a plasmonic environment. In Section 2, we start with a brief introduction to the crystal structure, optical properties, and electronic properties of 2D materials. In Section 3, we mainly present plasmonic structures, weak coupling, and strong coupling regimes, respectively. In Section 4, we review selected recent reports about the optical properties of 2D materials integrated with plasmonic structures. We also focus on hot electron photodetectors based on 2D materials integrated with plasmonic structures in this section. Finally, we summarize all the content of the review and present a little perspective on this novel field.

## 2. Basic Concept

### 2.1. Overview of 2D Materials

**Figure 1:**
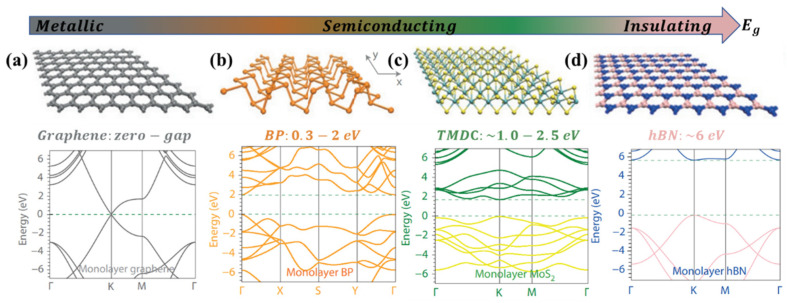
Schematic diagram of lattice structure and bandgap of respective 2D-layered materials [14] (**a**–**d**); Panel (**a**–**d**) adapted with permission from Ref. [14], copyright 2014, Nature Publishing Group, a division of Macmillan Publishers Limited. All Rights Reserved.

Graphene, which is composed of monolayer carbon atoms with a hexagonal honeycomb lattice by sp^2^ hybridization, is a gapless semimetal material with a linear dispersion relation near the Dirac point (shown in Figure 1a). A graphene monolayer has a light absorption rate of 2.3% in the infrared to visible spectrum range [39,40,41]. In addition, graphene has ultrahigh carrier mobility, reported up to 2 × 10^5^ cm^2^ V^−1^ s^−1^ at low temperature and high in-plane mobility of 1 × 10^4^ cm^2^ V^−1^ s^−1^ at RT [42,43] which allows for broadband light absorption or photodetection from terahertz (THz) to ultraviolet (UV) [39,44,45] with the ultrafast speed of picoseconds to nanoseconds [46,47,48].

TMDs have a sandwiched X-M-X layered structure, where M denotes a transition metal atom, and X represents a chalcogen atom [16]. Bulk TMDs are indirect semiconductors with distinct bandgaps, whereas monolayer TMDs have a direct bandgap, and their band energies range from 1 to 2.5 eV, as shown in Figure 1c. Several studies have shown that 2D TMDs not only possess controllable band gaps, but also have high carrier mobility on the order of 100–500 cm^2^ V^−1^ s^−1^ at RT [49,50,51]. These unique optical and electronic properties make 2D TMDs suitable for the application of high-performance photodetection and ultrathin field-effect transistors.

A few-layer black phosphorus (BP) is a recently reported 2D-layered material with a direct bandgap. As shown in Figure 1b, the atomic lattice of BP shows a honeycomb structure formed by the combination of one phosphorus atom and three adjacent phosphorus atoms. The electronic, phonon, and optical properties of few-layer BPs exhibit in-plane anisotropy due to the reduced lattice symmetry caused by the puckered-layer structure of BP [14,19,52,53,54]. A few-layer BP exhibits the characteristics of strong light absorption and PL emission. The carrier mobilities of few-layer BP [19] and single-layer BP [52,54,55] can reach up to 1000 cm^2^ V^−1^s^−1^ with ambipolar transport behavior at RT. BP has a controllable bandgap in the range of 0.3–2 eV that is layer–number dependent. The tunable bandgap few-layer BP is just between the energy bands of graphene and monolayer TMD; thus, it can be applied to photodetectors in the infrared to the visible range.

Seen in Figure 1d, hexagonal boron nitride (h-BN) has a graphene-like honeycomb lattice structure formed by alternating boron (B) and nitrogen (N) atoms. Boron nitride is a direct bandgap insulator with a large bandgap of 6 eV [55] that is suitable as a substrate to support other 2D materials to enhance device performance [56], especially in the case of graphene. The bandgap of h-BN lies in the mid-ultraviolet (MUV) ranging from 200 to 300 nm, enabling high-performance and deep UV photodetector [57,58,59,60,61].

### 2.2. Optical Properties

#### 2.2.1. Excitons in Monolayer TMDs

The optical properties of 2D semiconductors [62,63,64,65] are mainly manifested in excitons and valley-selective circular dichroism, etc. Thanks to layer quantum confinement, the screening effect in single-layer TMDs primarily leads to significant enhancement of electron–electron interactions that results in tremendous excitonic binding energy and oscillator strength [66]. It is reported that the exciton-binding energy of monolayer TMDs is in the range from tens to hundreds of milli-electron volts which is nearly two orders of magnitude larger than that in traditional semiconductors. Therefore, the excitons, trions [67], and higher-order bound states biexcitons with large binding energies in monolayer TMDs can remain stable even under thermal disturbance at RT. A large oscillator strength manifests in the strong exciton absorption because of the high screening effect in TMDs that can also facilitate strong coupling to light [68].

Monolayer TMDs such as MoS_2_ and WSe_2_ have a valley-dependent response to circularly polarized light [63,69,70,71,72,73,74] owing to the broken inversion symmetry in them. The direct bandgap transition of the monolayer TMDs occurs at the energy-degenerate K and K′ point of the 2D hexagonal Brillouin zone. The spin–orbit interaction leads to splitting of the valence band (greater than 150 meV) [75] which is divided into the sub-bands of spin-up and spin-down. Due to time-reversal symmetry, the Berry curvature at K and K′ valleys have to be opposite which leads to opposite orbital magnetic moments. Thus, the valley degree of freedom (DoF) of TMDs monolayer is locked with the spin DoF. In this way, the valley selection rule for optical transitions is formed. That is, the inter-band transition of the K valley couples with right circularly polarized light, while that of the K’ valley does with left circularly polarized light which brings a spin-like DoF to 2D exciton research [76,77,78].

#### 2.2.2. Nonlinear Optics in Monolayer TMDs

In addition to exciton physics, it is worth noting that 2D materials also exhibit rich nonlinear optical responses. The response polarization P(t) of an external electric field can be derived using Taylor’s expansion:

(1) $$P\left(t\right)=\chi^{\left(1\right)}E(t)+\chi^{\left(2\right)}E^{2}\left(t\right)+\chi^{\left(3\right)}E^{3}\left(t\right)+\ldots$$

where $\chi^{\left(n\right)}$ is the nth order nonlinear polarizability, and E(t) is the applied electric field strength. In terms of nonlinear efficiency, 2D materials all have excellent saturable absorption strengths. Compared with graphene and BP, monolayer TMDs have more advantages in nonlinear optics. Thanks to their inversion asymmetry and large excitonic resonance, second-order nonlinear polarizability $\chi^{\left(2\right)}$ of TMDs monolayers is large which results in a large second harmonic generation (SHG) process [11,64,79,80,81,82]. As a vital branch of modern optics, nonlinear optics has important applications in generating ultrashort pulses, laser spectrum conversion, ultrafast optical switching, and all-optical signal processing [11,83]. In addition, other optical properties such as the valley Hall effect [76] and dark excitons [84,85] of monolayer TMDs have also attracted extensive attention of many research groups.

## 3. Plasmonic Environments

Plasmonic metallic structures have the characteristics of subwavelength confinement, electromagnetic field enhancement, and tunable far-field radiation energy, that can strengthen the interaction of 2D materials with light [86,87,88,89,90], thus enhancing optical absorption and emission. Generally speaking, the plasmonic environment has the following types: plasmonic nanoantenna, plasmonic nanocavity, plasmonic array, plasmonic waveguide, plasmonic metamaterials, etc. In this section, we discuss plasmonic environments that are relevant to plasmonic effects, plasmonic nanostructures, and coupling regimes with 2D materials.

### 3.1. Plasmonic Effects and Plasmonic Nanostructures

#### 3.1.1. Plasmonic Effects

SP is a fundamental quasiparticle in solid state physics that is defined as coherent free electron collective oscillation in metal. According to the form of propagation, SP can be divided into two categories: localized surface plasmons (LSP) localized near the metal particles and surface plasmon polaritons (SPP) propagating on metal surface [91,92].

Based on Maxwell’s equations with the continuous boundary conditions of electromagnetic field interface, the dispersion relationship of SPP is given by the following equation:

(2) $$k_{SPP}=\frac{\omega }{c}\sqrt{\frac{\varepsilon_{d}\varepsilon_{m}(\omega )}{\varepsilon_{d}+\varepsilon_{m}(\omega )}}$$

where $\varepsilon_{d}$ is the relative permittivity of dielectric, and $\varepsilon_{m}(\omega )$ is the relative permittivity of the metal as a function of frequency of the incoming light. $\varepsilon_{m}(\omega )$ can be described by $\varepsilon_{m}(\omega )=1-\omega_{p}^{2}/\omega^{2}$ using the Drude model in which $\omega_{p}$ is the plasma frequency of bulk. In low frequency range, $k_{SPP}\approx \omega /c\sqrt{\varepsilon_{d}}$ of SPP is close to the dispersion line of the incident light in the medium, which shows its propagating properties. When the wave vector of SPP meets $k_{SPP}\to \infty$, the angular frequency can be expressed as $\omega =\omega_{sp}=\omega_{p}/\sqrt{1+\varepsilon_{d}}$. When the frequency of incident light approaches $\omega_{sp}$, the wavelength of SPP goes near zero, which indicates that the electric field decays rapidly in a normal direction of the interface and is tightly bound on the metal surface. The dispersion curve of SPP represents that the momentum of SPP is always greater than that of incident light, which means that the excitation of SPP needs a specific momentum compensation condition. Prism coupling and grating configuration [93] are two typical methods to achieve the phase matching between SPP and incoming light. The SPP propagating at the metal–dielectric interface is actually a transverse magnetic (TM) wave with the magnetic field perpendicular to the direction of propagation. In terms of physical nature, a non-zero electric field component perpendicular to the interface makes electrons accumulate on the metal surface, and then these electrons move collectively under the action of the tangential component of the electric field. Therefore, an electric field perpendicular to interface decays exponentially, leading to a highly localized and enhanced electromagnetic field near the metal surface. A large number of novel metal nanostructures have been computationally simulated and experimentally fabricated, and the corresponding plasmonic properties have been studied and exploited in other materials [21,93].

LSP is another type of plasmonic effect, which is mainly attributed to confined electrons on the surface of metal nanoparticles (NPs) [94]. The modes of non-propagating plasmons arise mainly from interactions between incident light and subwavelength metal NPs. Mie theory can be utilized for analyzing the resonant excitation modes of nanospheres by solving Maxwell’s equations. The dispersion relation of LSP obtained from the Mie theory is not continuous but can be determined by discrete resonance modes with different orders. Generally speaking, localized surface plasmons resonance (LSPR) corresponds to the maximum values of polarizability α and scattering absorption efficiency. Thus, the total optical responsivity is described by the extinction cross section $\sigma_{ext}$, which is numerically the sum of absorption $\sigma_{abs}$ and the scattering cross section $\sigma_{scat}$. When nanoparticle size is much smaller than the incident wavelength, Mie theory can ignore the contribution of the high-pole resonance modes. In this situation, the metal nanospheres can be regarded as a simplified dipole using an electrostatic approximation tool, and polarizability α can describe a scattering and absorption cross section as the following relation:

(3) $$\sigma_{abs}=\frac{k^{4}}{6\pi }{\left|\alpha \right|}^{2}=\frac{8\pi }{3}k^{4}R^{6}{\left|\frac{\varepsilon_{m}(\omega )-\varepsilon_{d}}{\varepsilon_{m}(\omega )+2\varepsilon_{d}}\right|}^{2}$$

(4) σscat=kIm{α}=4πkR3Im{εm(ω)−εdεm(ω)+2εd}

The dipole scattering and absorption cross section simultaneously reach a maximum when the real part of permittivity satisfies Re[εm(ω)]=−2εd. In this situation, LSPR occurs and the electric field near the metal surface is greatly strengthened. The electric field enhancement factor is mainly determined by the material composition, that is, the shape and size of metal NPs [95]. The distribution, enhancement, and far-field emission of a LSP can be flexibly tuned by designing the geometry of metal NPs. If these NPs are assembled into a nanoarray, interaction between NPs can further enhance the near field and reduce the SP peak width [96].

#### 3.1.2. Plasmonic Nanostructures

**Figure 2:**
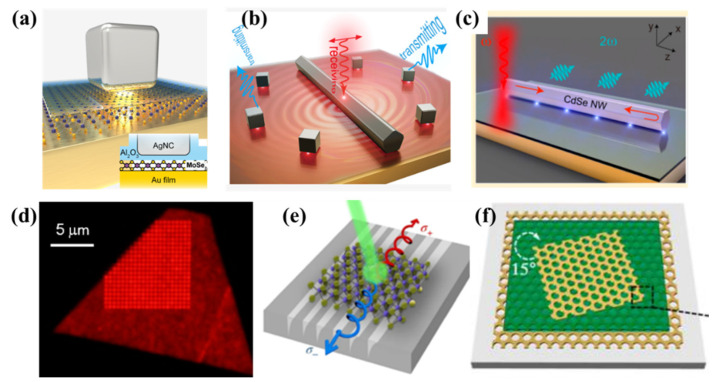
Schematics of plasmonic environments: (**a**) schematic of plasmonic nanocavity of silver nanocube over a gold substrate, separated by 2D semiconductor [103]; (**b**) schematic of matched plasmonic nanoantenna pair [110]; (**c**) cchematic diagram of plasmonic waveguide consisting of semiconductor nanowires and gold films [113]; (**d**) sample microscopic image between monolayer MoSe_2_ and plasmonic hole array [111]; (**e**) illustration of MoS_2_ monolayer coupling to metasurface consisting of subwavelength asymmetric grooves [114]; (**f**) Moiré chiral metamaterials (MCMs) is stacked with two layers of identical Au nanohole arrays and a dielectric spacer layer [115]; panel (**a**) adapted with permission from Ref. [103], copyright 2019, American Chemical Society; panel (**b**) adapted with permission from Ref. [110], copyright 2020, American Chemical Society; panel (**c**) adapted with permission from Ref. [113], copyright 2019, American Chemical Society; panel (**d**) adapted with permission from Ref. [111], copyright 2016, American Chemical Society; panel (**e**) adapted with permission from Ref. [114], copyright 2019, Liuyang Sun et al., under exclusive license to Springer Nature Limited; panel (**f**) adapted with permission from Ref. [115], copyright 2018, American Chemical Society.

The abundance of plasmonic nanostructures can satisfy the requirements of various operating systems. A plasmonic nanocavity, composed of a metallic nanostructure represents one of the focuses of the current optical cavities and is a conventional means of strengthening the light–matter interaction at the nanoscale. The quality factor (Q) and mode volume (V) are two main effective parameters for evaluating the characteristics of the optical cavity. For many specific systems, a higher Q and a smaller V are necessary to obtain an enhancement factor of light–matter interaction [97,98]. Designing the geometry of a metallic nanostructure, the local electromagnetic fields of SP can be compressed to a sharply enhanced region on the subwavelength scale to construct some nanocavity that is much smaller than the wavelength of plasmonic resonance [26,99,100,101,102]. Figure 2a shows one example of plasmonic nanocavities which is silver nanocubes over a gold substrate separated by a 2D semiconductor [103] for enhancing Raman and fluorescence intensities. The ultrasmall mode volume of the plasmonic cavity can be 3–4 orders of magnitude smaller than (λ/n)^3^ [26]. The strong focusing ability of the plasmonic nanocavity on electromagnetic fields provides an unprecedented possibility for manipulating the light–matter interaction [26]. Linewidths of cavity modes are plagued by plasmonic loss, that is, the rapid dephasing of SP that transfers their energy to single-electron excitations [104], drastically decreasing the nanocavity photons’ lifetime and the value of Q. However, researchers have now started to embrace the loss-enabled applications of plasmonics [105] such as thermo-plasmonics [23,106,107,108], surface imaging [109], and hot carrier generation for photochemistry and photodetection [24,28,29,30,31,32]. For plasmonic nanoantennas, shown in Figure 2b, the receiving nanoantenna is composed of Ag nanowire/spacer/metal mirror [110] which ensures high light–plasmon conversion efficiency. The transmitting antennas consist of silver nanocube/spacer/metal mirror whose impedances are matched to free-space photons and propagating surface plasmons. Receiving and transmitting nanoantennas are spatially separated, but the SPP propagating on the metal film enables signal transferring between them. The resonance-matched nanoantenna pairs demonstrate how to achieve enhanced Raman spectral detection with separable excitation and collection light. In Figure 2c, the SHG signal was enhanced owing to the overlap of guided modes in the hybrid plasmonic waveguide. A larger absorption coefficient can be further attained by utilizing a periodic Ag plasmonic hole array with stronger plasmonic resonance enhancement (Figure 2d) that makes the linewidth of the exciton narrower and strengthens the intensity of PL [111]. In addition, Ritesh Agarwal et al. proposed a strongly coupled exciton–plasmon system consisting of monolayer MoS_2_ and Ag nanodisk arrays which provided a novel idea to design a SPP device [112].

Metasurfaces or metamaterials are another class of plasmonic nanostructures [116,117,118]. Optical metamaterials consisting of nanoscale building blocks of metal, semiconductor, or dielectric meta-atoms provide an excellent photonic environment for integrated materials. Plasmonic metamaterials are commonly fabricated by some metals such as gold (Au), silver (Ag), aluminum (Al), and platinum (Pt) which can improve light–matter interactions ranging from midinfrared (MIR) to UV wavelengths. The first optical metasurface is made of periodic V-shaped metal arrays on a dielectric substrate which can be used to tune the phase of transmitted light [119]. In addition to the periodic V-shape plasmonic metasurface, another type of plasmonic metasurface composed of periodic Au nano-slits with different in-plane rotation angles can manipulate different chirality light emission in monolayer TMDs [120]. In Figure 2e, the periodic plasmonic asymmetric groove metasurface enables the sorting and spatial separation of valley-polarized excitons from MoS_2_ monolayers at K and K′ points [114]. As shown in Figure 2f, the MCMs is a chiral metamaterial regulated by a spacer layer, which can greatly modulate the circular dichroism spectrum.

### 3.2. The Weak and Strong Coupling

In the previous section, we discussed the integration of 2D materials with plasmonic nanostructures, including plasmonic cavities [121,122], plasmonic waveguides [123], plasmonic arrays [111], and plasmonic metasurfaces [119,124] that enhance the light–matter interactions and release 2D materials’ excellent capabilities. Due to the near-field enhancement effect of SP, the interaction between excitons in semiconductors and light is naturally enhanced. Among them, according to the strength of the light–matter interaction, it can be roughly divided into the weak coupling and the strong coupling regime, respectively. The optical properties of the composite system of 2D materials and plasmonic nanostructures are quite different in these two different coupling types [125,126].

#### 3.2.1. Weak Coupling Regime

In the weak coupling regime, due to the low coupling strength, the rate of energy exchange is less than the decay rate of each coupling component; thus, the energy of the system is exhausted before the energy exchange reaches a complete cycle. In the hybrid system of 2D materials coupled with plasmonic structures, the decay rate of exciton will become faster because of the influence of SP. This phenomenon that the spontaneous emission rate of 2D materials is modulated by changes in the external environment is called the Purcell effect. It can be quantitatively described by the Purcell Factor, and its expression can be written as [98]

(5) $$F_{p}\equiv \frac{\gamma_{ex}}{\gamma_{ex}^{0}}=\frac{3}{4\pi^{2}}{\left(\frac{\lambda }{n}\right)}^{3}\frac{Q}{V_{eff}}$$

where $F_{p}$ is the Purcell factor, $\gamma_{ex}$ is the total exciton decay rate in the 2D material, $\gamma_{ex}^{0}$ is the radiative decay rate of the exciton in free space, λ is the resonance wavelength of SP, n is the refractive index, Q is the quality factor of the resonator, and $V_{eff}$ is the effective mode volume. The Purcell effect explains the interaction of SP and excitons. The presence of SP can selectively enhance the spontaneous decay rate of 2D materials at certain frequencies, owing to the tunable resonance modes of SP. The energy of excitons in the 2D materials can only be transferred to the photons [127,128]. It can be known from the Purcell factor that maximizing $Q/V_{eff}$ is beneficial to improve the efficiency of excitonic emissions. The Purcell effect can not only enhance PL but also strengthen other optical responses such as phosphorescence and Raman scattering [26]. Therefore, tuning weak coupling is the most important way to control the optical properties of 2D materials which plays a crucial role in lasers, light-emitting diodes (LED), ultra-sensitive light sensing, and quantum information processing [26,127,128].

#### 3.2.2. Strong Coupling Regime

In a strongly coupled system, there is at least one cycle of coherent energy exchange. The higher coupling strength brings more cycles of this energy exchange, resulting in more Rabi oscillation cycles. The Rabi oscillation in the time domain leads to Rabi splitting in the frequency domain [127,128,129,130]. As a result, new eigenstates with prominent anti-crossed states called exciton–polaritons (EPs) states of partial photons and partial excitons are formed in the specific energy dispersion diagrams [127,128,129,130,131].

In most cases, the linewidth of the cavity photon is relatively large and is directly inversely proportional to Q. Strong coupling can be achieved when Q/V_eff_ becomes larger which means the rate of coupling strength and the linewidth of cavity photon are larger at the same time [127,128,129,130]. EPs, possessing dual properties of photons and excitons, exhibit the high propagation properties of photons, an ultra-light effective mass, multiple photon degrees of freedom, strong nonlinear interactions of excitons, and sensitive optoelectronic responses. The 2D materials integrated with a cavity system in the strong coupling region have pushed the research to the frontier fundamental problems and the devices of application of nanophononics [125,126,131].

## 4. Combining Plasmonic Structure with 2D Materials

To exploit the advantages of the optical properties of 2D materials, one of the critical methods is to couple 2D materials with plasmonic nanostructures such as nanocavities [121,122] and metamaterials [132] which exhibit more excellent capabilities, for example, improving fluorescence emission and Raman intensity [133,134], manipulating the valley DoF of excitons in 2D materials [135], enhancing nonlinear optical signals [136,137], and obtaining Eps [138] states. In this section, we will review the many enhanced optical functionalities resulting from the combination of 2D materials and plasmonic nanostructures.

### 4.1. Enhancement of PL Emission

**Figure 3:**
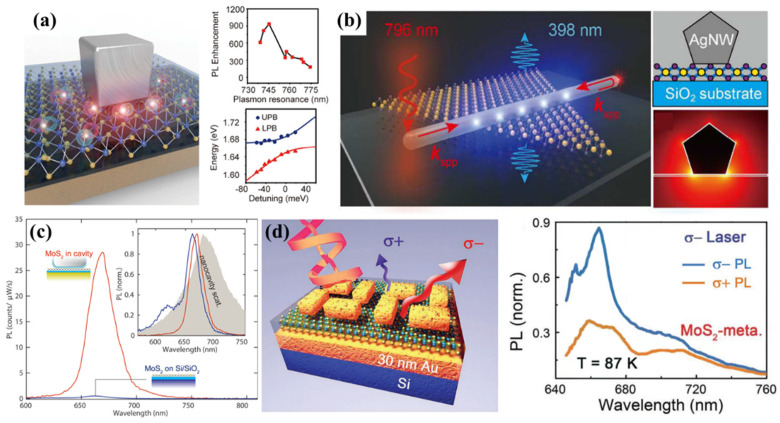
2D materials integrated with plasmonic environments: (**a**) The nanocavity composed of silver nanocubes and mirror Au film can effectively increase the PL intensity of monolayer WSe_2_ [148]; (**b**) The SHG signal was enhanced by the hybrid optical plasmonic waveguide consisting of a 1D Ag nanowire and a monolayer MoS_2_ sheet [123]; (**c**) Under the action of the plasmonic resonator, MoS_2_ enhances light absorption, thereby improving QY [143]; (**d**) Valley-polarized PL emission of MoS_2_ was controlled by the metal–dielectric–metal plasmonic chiral metamaterials [149]; Panel (**a**) adapted with permission from Ref. [148], copyright 2018, American Chemical Society; Panel (**b**) adapted with permission from Ref. [123], copyright 2017, American Chemical Society; Panel (**c**) adapted with permission from Ref. [143], copyright 2015, American Chemical Society; Panel (**d**) adapted with permission from Ref. [149], copyright 2018, WILEY-VCH Verlag GmbH & Co. KGaA, Weinheim.

Compared with the radiative lifetime of the nanosecond scale for TMDs excitons, their non-radiative lifetime is short at hundreds of picoseconds [17,139] which makes the PL efficiency very low at RT. Earlier studies on improving PL emissions focused on the integration of a nanocavity with MoS_2_ [10,140,141]. A plasmonic nanocavity can achieve subwavelength localization and electromagnetic field energy enhancement by confining SPP to the metal–dielectric interface which achieves Purcell enhancement of TMDs over the visible to infrared spectrum range [142]. As shown in Figure 3c, a monolayer MoS_2_ was placed in a plasmonic nanocavity composed of a silver nanocube and Au substrate [143], where PL emission increased by two orders of magnitude due to the Purcell enhancement caused by the extremely small V_eff_ of the nanocavity [143]. As shown in Figure 3a, the Xu group studied the behavior of excitons in 2D materials under the action of local fields in the nanocavity and realized the Rabi splitting of plasmons and excitons in 2D quantum systems. By comparing the energy of Rabi splitting and the linewidth of the excitons, it is demonstrated that the system is in the critical coupling region between weak coupling and strong coupling. The PL emission intensity in the hybrid system of monolayer WSe_2_ and plasmonic nanocavity is enhanced 1700 times. They first proposed the concept of plasmon–exciton enhanced fluorescence which is the reason for the high PL magnification. This work is different from the plasmon-enhanced PL phenomenon in the weak coupling region. Plasmonic metamaterials exhibit a different optical dispersion relationship from resonant cavities [144,145,146] which can highly strengthen the photonic density of states (PDOS) over a broad frequency range rather than looking to maximize Q/V_eff_. A hybrid structure of a metasurface combined with PCC can also enable directional PL emission of single-layer TMDs deposited on the hybrid metasurface [147].

### 4.2. Enhancement of Valley-Selective Circular Polarization Photoluminescence

The valley DoF of TMD materials can be used for information storage and information processing which has received extensive attention. However, 2D materials have pronounced phonon-assisted inter-valley scattering at RT which reduces the fidelity of valley-polarized PL signals. To achieve high photoinduced circularly polarized luminescence at RT, it is crucial to control valley dynamics. Plasmonic metamaterials typically exhibit large chiral features. The chiral response of specific plasmonic metamaterials can control the valley DoF of 2D material excitons and valley-selective circular PL emission [135,138,150]. As shown in Figure 3d, the monolayer MoS_2_ is inserted into a chiral nanocavity composed of a gold film and a chiral metamaterial [149]. The chiral cavity controls the valley-polarized far-field exciton emission of monolayer MoS_2_ because the chiral Purcell effect changes the spontaneous emission rate in different valleys (K and K′ valleys). Another report demonstrated that a monolayer of MoS_2_ in a chiral plasmonic nanocavity achieves up to 48.7% circularly polarized PL emission at RT [135] due to the huge chiral Purcell effect by the degeneracy of the circularly polarized local density of states in the plasmonic nanocavity. These studies provide a possible route to develop various light-emitting devices based on valley coding at RT.

### 4.3. Enhancement of Nonlinear Optical Response

The special lattice structure with inversion asymmetry and large exciton energy of monolayer TMDs usually lead to a large SHG process. However, the limited light absorption due to atomically thin thickness results in low-frequency conversion efficiency. A plasmonic structure can be used to further enhance the nonlinear effects because of its strong light-binding ability. Figure 3b shows that a hybrid system formed by a single Ag nanowire (AgNW) combined with monolayer MoS_2_ is capable of generating the SHG signal with axial collimation but transverse divergence by using remotely excited SP [123]. The strategy of exciting the SHG process reveals unique features of enhanced nonlinear optics in one-dimensional plasmonic waveguides. Zhuo et al. observed a 7000-fold enhancement of a SHG signal in monolayer WSe_2_ coupling with a sub-20 nm wide Au grating deposited on flexible polydimethylsiloxane (PDMS) substrates [137]. Guo et al. demonstrated in 2020 that a three-order-of-magnitude improvement in mixing conversion efficiency of a WS_2_ monolayer is achieved under the excitation of counter-plasmon propagation due to an enhancing overlap of the waveguide modes and nonlinear WS_2_ in the temporal and spatial scales. In 2019, the Qiu group used a gold metasurface with birefringence properties to generate two types of circularly polarized light that excited the WS_2_ monolayer on a metasurface to emit a SHG signal with different circular polarization [120].

### 4.4. Strong Coupling Regime between 2D TMDs and Plasmonic Nanostructure

**Figure 4:**
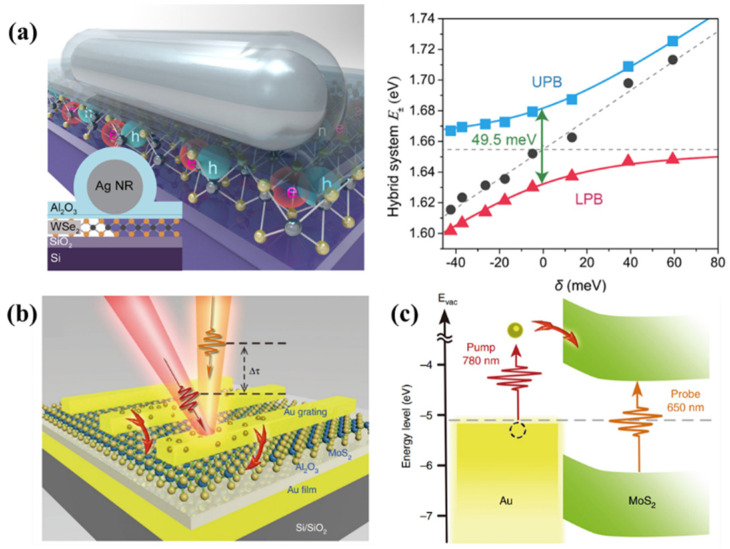
(**a**) The monolayer WSe_2_ is coupled with the plasmonic cavity to obtain the EPs state [153]; (**b**) MoS_2_ monolayer was inserted in a Au gating–insulator–Au film sandwich structure [156]; (**c**) Transfer of hot electrons between a MoS_2_ monolayer and a Au array was measured by pump-probe technology [156]; Panel (**a**) adapted with permission from Ref. [153], copyright 2017, American Chemical Society; Panel (**b**,**c**) adapted with permission from Ref. [156], copyright 2019, Hangyong Shan et al.

Because of the large oscillator strength and binding energy of 2D TMDs’ excitons, EPs in 2D materials integrated with plasmonic structures can be formed at RT in the regime of strong coupling [125,126,131]. Plasmonic nanocavities provide a special experimental system for strong coupling effects in 2D materials [151]. As described above, SP can greatly improve the coupling strength of 2D excitons and cavity photons due to their ultra-small mode volume V. Although its quality factor Q is often small, many plasmonic nanocavities can obtain relatively high Q/V, so it is easy to achieve strong coupling effects. At present, plasmonic nanocavities strongly coupled with 2D TMDs have also made rapid progress in various forms [68,152]. In Figure 4a, a plasmonic nanocavity composed of silver nanorods exhibits an anticross-form EPs energy state in spectroscopic measurements of dark field scattering [153]. To date, plasmonic cavity systems have been used to seek the strong coupling of 2D TMDs [68,154,155]. Being similar to the plasmonic nanocavity, the photonic mode based on plasmonic arrays also has a very small cavity volume V which enhances the strong coupling effect. The photon modes of a typical plasmonic array and the 2D excitons coupled to the k-space reflectance spectrum and the PL spectrum show a very obvious anticrossover relationship that confirms the strong coupling effect as shown in Figure 2d [111]. Strong coupling effects have also been found in various forms of plasmonic arrays [112]. As shown in Figure 4b, the strong coupling effect between the LSP and the propagating SP mode is realized by constructing a metal–insulator–metal sandwich structure which improves the efficiency of generating and transferring hot electrons. The non-radiative relaxation loss of SP generates hot electrons with high kinetic energy that can be widely used in photovoltaic devices, photocatalysis, and nonlinear optics. The direct observation of the ultrafast transfer of hot electrons was achieved by using femtosecond pump–probe technology as shown in Figure 4c. The experiment showed that the hot electron transfer time of SPP was about 40 fs, and the maximum external quantum efficiency reached 1.65%. The strong coupling effect can effectively enhance the hot-electron transfer efficiency which is of great significance for the application of SP hot electrons.

## 5. 2D Material-Based Photodetectors with Plasmonic Nanostructures

Since the discovery and fabrication of 2D materials, photodetectors based on these materials have been intensively studied. Conventional photodetection includes photoconductive [157], photovoltaic [158,159,160], phototransistor [161], and pyroelectric devices [160]. High-performance photodetectors are widely used in many fields of daily life, including imaging [162], surveillance [163], telecommunication [164], and more. However, the weak light absorption of 2D materials limits their application on photodetectors. Currently, including but not limited to integrated plasmonic structures, they are used to increase the interaction of light and matter in photodetectors of 2D materials. Based on the theory of SP hybridization, metal nanostructures can be designed for highly absorbing interfaces with the resonant wavelength. At the same time, the detection wavelength, incident light intensity, and polarization can be regulated by using decoration of plasmonic NPs or nanoarrays [41,165,166,167]. For example, a photodetector based on multilayer MoS_2_ can be improved by increasing the optical resonance absorption and enhancing the photocurrent, owing to the presence of Au nanostructures [168]. For another example, the absorption rate of graphene is increased by 20 times after coupling Au plasmonic nanoantenna [40]. In addition, the LSPR of metal nanostructures induced by an external light field will generate hot electrons after the decay [169,170]. This can be combined with some 2D materials with high electron mobility to achieve applications in photodetection and other aspects [92]. At present, the hot electron mechanism of LSPR has been applied to photodetectors based on graphene [171] and TMDs [90]. In this section, we mainly discuss how hot electrons can dynamically control the photocurrent in photodetectors of 2D materials integrated with plasmonic structures.

Table 1 represents the different photodetector parameters in MoS_2_ or graphene layers integrated with plasmonic nanostructures or nanoarrays discussed in this review. The results presented here represent that enhancement of responsivity in these photodetectors resulting from plasmonic resonance enhancement or hot electron injection.

### 5.1. Hot Electron Photodetectors Based on Graphene

**Figure 5:**
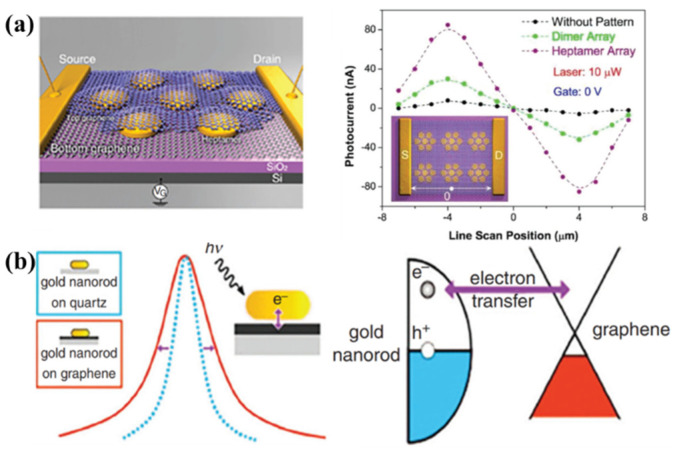
2D materials: (**a**) sandwich structure consisting of two monolayer graphene and Au nanontennas (**left panel**); photocurrent measurement response curves for different regions on sandwich structure (**right panel**) [171]; (**b**) the coupling of the Au nanorods to the graphene monolayer increases the linewidth of the plasmonic mode, predicting that the hot electrons take about 160 fs to transfer (**left panel**); schematic diagram of the transfer process of hot electrons between graphene and Au nanorods [173]; panel (**a**) adapted with permission from Ref. [171], copyright 2012, American Chemical Society; panel (**b**) adapted with permission from Ref. [173], copyright 2013, American Chemical Society.

The photocurrent of 2D materials photodetectors coupled with plasmonic nanostructures can be improved in the plasmonic resonance wavelength range [12,87]. As shown in Figure 5a, the photodetector was based on the sandwiched nanostructure consisting of two monolayer graphene and Au nano heptamer, where the photocurrent was increased by nearly three orders of magnitude. The reason is that the hot electrons generated by the non-radiative decay of the SP modes were combined with electrons of high mobility of the graphene [171]. In that study, the excitation light was scanned in a line between the source and the drain electrodes, and the corresponding local photocurrent was investigated. The antisymmetric curve characteristic of the photocurrent indicates that the existence of Au nanoantennas provides multiple localized spots of enhanced electromagnetic energy, resulting in a higher density of hot electrons than ever before. As shown in Figure 5b, the generated hot electrons can directly enter graphene without an external electric field, owing to the zero bandgaps of graphene [173]. The transfer time of hot electrons between them has been predicted at about 160 fs by comparing the linewidths of the resonant SP modes with and without graphene. Researchers mainly focus on the study of the injection process of hot electrons generated by LSPR in the plane of graphene in previously cited reports. The injection process of hot electrons in the vertical direction needs to be realized by the tunneling effect because of the existence of anisotropy in 2D materials. The Fang group successfully detected the hot electron tunneling current in its vertical direction by coating Au NPs on the monolayer graphene [93]. The photoelectric responsivity reached the strongest at the resonant modes of Au NPs under supercontinuum laser excitation which confirms that hot electrons relaxed by LSPR can tunnel through the graphene monolayer to reach the back electrode. The photocurrent became larger with the increase of voltage and light intensity by applying a bias voltage between the top and back electrodes and increasing the intensity of incident light. When the incident light intensity increased to a certain value, a built-in electric field was formed between the Au nanostructure and graphene which promoted the recombination of carriers and saturated the photocurrent. In addition, the tunneling effect of hot electrons became weaker with the increase of the number of layers, by transferring the graphene monolayer multiple times to fabricate a multilayer graphene barrier which can be explained using excited-state lifetimes and their dynamic decay processes in multilayers graphene barriers.

### 5.2. Hot Electron Photodetectors Based on MoS_2_

**Figure 6:**
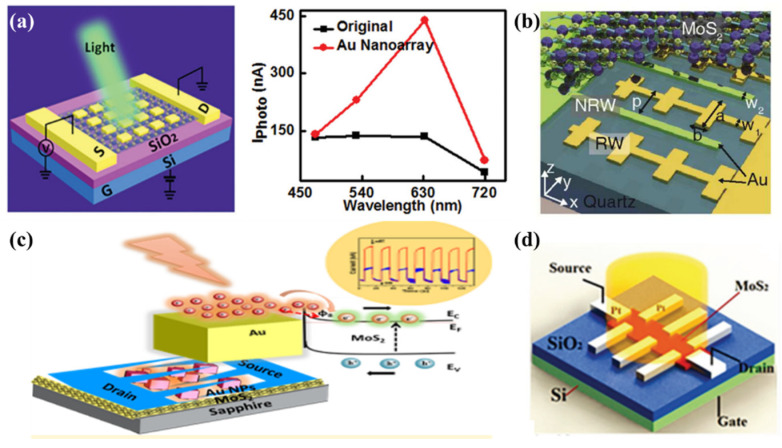
(**a**) (**Left**) schematic of a multilayer MoS_2_ phototransistor coupled to metal nanoarrays. (**Right**) Photocurrent as a function of excited wavelength [87]; (**b**) schematic illustration of photodetector consisting of bilayer MoS_2_ and Au nanoarray [90]; (**c**) photoresponsivity of bilayer MoS_2_ photodetection was enhanced by a factor of 32 when MoS_2_ was deposited by Au NPs [133]; (**d**) photoconductivity based on bilayer MoS_2_ coupled with Pt nanostrips [176]; panel (**a**) adapted with permission from Ref. [87], copyright 2015, WILEY-VCH Verlag GmbH & Co. KGaA, Weinheim; panel (**b**) adapted with permission from Ref. [90], copyright 2015, American Chemical Society; panel (**c**) adapted with permission from Ref. [133], copyright 2020, American Chemical Society; panel (**d**) adapted with permission from Ref. [176], copyright 2017, WILEY-VCH Verlag GmbH & Co. KGaA, Weinheim.

Photodetector devices based on TMDs have lower photoresponsivity. Based on the LSPR effect, 2D materials integrated with metal NPs or nanoarrays can effectively enhance the photocurrent of the devices. As shown in Figure 6a, the photocurrent response of the multilayer MoS_2_ transistor is strengthened by a factor of three under the plasmon resonance enhancement of periodic Au nanoplates. A one-fold enhancement of photocurrent was observed when Au NPs were evenly deposited onto multilayer MoS_2_. These observations resulted from the improvement of light-harvesting near the metal NPs [87]. MoS_2_ has also emerged as an ideal acceptor for hot electrons. In a recent study, ultrafast transfer of plasmonic hot electrons occurred in the structure of monolayer MoS_2_ integrated with a Au nanoantenna within 200 fs [172]. Furthermore, the exciton energy of MoS_2_ was enhanced and reradiated under the interaction of LSPR. The multilayer MoS_2_ had an indirect bandgap, compared to the single-layer MoS_2_ with a direct bandgap. Coupled with localized plasmons, multilayer MoS_2_ could easily form a lower Schottky barrier which would result in hot electrons entering the R-point band instead of the band of K point [174,175]. As shown in Figure 6b, Wang et al. in 2015 reported a photodetector of bilayer MoS_2_ coupled with a metal nanoantenna array [90]. Hot electrons were injected into MoS_2_ through the Schottky barrier between MoS_2_ and metal which resulted in a photoexcited current below the bandgap and extended the photoresponse into the NIR region. Moreover, the injection of hot electrons led to 10^5^ enhancement of photo gain of MoS_2_ and a photoresponsivity of 5.2 A W^−1^ that was much higher than that of silicon-based photodetectors with hot electrons transfer. As shown in Figure 6c, the Yen research group, in 2020, proposed one photodetection based on bilayer MoS_2_ decorated by plasmonic NPs [133]. The blue shifts of the bandgap of the bilayer MoS_2_ stem from the effect of the plasmonic strain. A beneficial tailoring of band structure and hot electrons injection enhanced the photoresponsivity of the photodetector by 32 times. Compared with conventional metals such as Au and Ag, Pt may be more beneficial for the design of plasmonic optoelectronics because of its broader spectral response and higher photo sensitivity [176]. Figure 6d shows photoconductivity fabricated by integrating Pt nanostrips with bilayer MoS_2_. The photocurrent yielded three orders of enhancement with the excitation of 532 nm light, when bilayer MoS_2_ coupled to Pt nanoribbons. These results are owing to the more efficient transfer of hot electrons by Pt nanoribbons compared to Al and Au.

## 6. Conclusions and Perspectives

In summary, the advancement of the frontier in 2D material coupled with a plasmonic have been reviewed in this paper. First, we summarized plasmonic environments into three aspects: types of plasmonic nanostructures, weak coupling regime, and the strong coupling regime, after briefly discussing the basic physical properties of 2D materials. Then, plasmonic nanostructures integrated with 2D materials were introduced, including a plasmonic cavity, a plasmonic waveguide, a plasmonic array, and plasmonic metasurfaces that enhance the light–matter interactions and release 2D materials’ excellent capabilities. Finally, we reported 2D material photodetectors integrated with plasmonic nanostructures, mainly focusing on graphene and MoS_2_. The integration of 2D materials with plasmonic nanostructures provides many advantages such as enhancing QY, manipulating the valley DoF of 2D materials excitons, improving nonlinear optical signal, and forming EPs states. Plasmonic techniques relevant to 2D material-based photodetectors exhibit a high photo gain and photoresponsivity due to resonant absorption enhancement and plasmonic hot electron conversion.

Finally, it is worth envisioning some follow-up research in this direction. Apart from plasmonic nanostructures, topological photonic crystals (TPCs) with resonant waveguide modes have emerged as the most prevalent topics in nanophonics which allow for nonlocal light–matter interactions. By using TPCs, many exciting properties can be modulated, such as photonic valley Hall effect [177], third-harmonic generation [178], and topological valley transport [179]. Recently, helical topological exciton-polaritons have been observed through monolayer WS_2_ flake coupling with the topological photonic crystal edge state [180]. In addition, the recent emergence of novel optical cavity forms such as photonic crystal bound states in the continuum and BICs state may provide some new ideas for the topological optics of 2D semiconductors integrated with optical cavities [181,182,183]. In particular, BICs are states with theoretically infinite high-quality factors despite the availability of a radiative continuum at the same frequency. Symmetry-protected BICs based on all-dielectric metasurfaces have been demonstrated to enhance wide applications of integrated 2D materials, such as sensors, low threshold lasers, vortex lasers, and harmonic generation.

## Figures and Tables

**Table 1:** Summary of plasmonic enhancement photodetectors in MoS_2_ and graphene. Plasmonic resonance (PR) enhancement; Hot electrons (HE) injection.

System	Responsivity/Photocurrent	Mechanism	Wavelength/nm	Reference
Monolayer MoS_2_	Photocurrent dichroism up to 60%	PR	532, 633	Eginligil et al. (2015) [169]
MoS_2_ +Au NPs/nanoarrays	Two/three-fold enhancement at 632 nm	PR	477–732	Miao et al. (2015) [87]
Bilayer MoS_2_ + Au nanoarrays	5.2 A W^−1^ at 1070 nm 1.1 × 10^5^ A W^−1^ at 532 nm	HE	532, 1070–1150	Wang et al. (2015) [90]
Bilayer MoS_2_ + Au NPs	790 μA/W, 32 times enhancement	Plasmonic strain; HE	532, 634	Sriram et al. (2020) [134]
Bilayer MoS_2_ + Pt nanostrips	Three-orders of enhancement at 532 nm	HE	325, 532, 980	Kumar et al. (2017) [172]
Graphene + Au nanoarrays	20 times enhancement at 514 nm	PR	514, 633	Echtermeyer et al. (2011) [40]
Graphene + Au heptamers	Enhancement of 800%	PR; HE	650–1000	Mubeen et al. (2012) [173]
Graphene + Au NPs	0.16 nA/μW at 710 nm	Tunneling effect; HE; PR	600–800	Du et al. (2017) [94]
